# Transcriptomic analysis of skin biopsies in Prurigo nodularis patients: with and without atopic dermatitis

**DOI:** 10.3389/fimmu.2025.1572413

**Published:** 2025-11-05

**Authors:** So Yeon Lee, Ji Young Um, Han Bi Kim, Hyun-Woo Yang, In Suk Kwak, Bo Young Chung, Chun Wook Park, Hye One Kim

**Affiliations:** ^1^ Department of Dermatology, Hallym University Kangnam Sacred Heart Hospital, Seoul, Republic of Korea; ^2^ Upper Airway Chronic Inflammatory Diseases Laboratory, Korea University College of Medicine, Seoul, Republic of Korea; ^3^ Department of Anesthesiology and Pain Medicine, College of Medicine, Hallym University, Hangang Sacred Heart Hospital, Seoul, Republic of Korea

**Keywords:** Prurigo nodularis (PN), atopic dermatitis (AD), chronic pruritus, differentially expressed genes (DEGs), Th2 inflammation, skin barrier dysfunction

## Abstract

**Background:**

Nodular dermatitis (PN) is a severely itchy chronic skin disease with symmetrically distributed nodules, often linked to an atopic background in some patients. However, the pathogenesis of PN with atopic dermatitis remains unclear.

**Objective:**

The objective of this study is to compare the transcriptomes from skin biopsies of prurigo patients with and without atopic dermatitis, aiming to identify unique gene expression patterns and gain insights into the molecular mechanisms underlying Atopic dermatitis Prurigo (ADP) and Non-Atopic dermatitis Prurigo (NADP).

**Method:**

We conducted transcriptome analysis to compare gene expression between normal controls and atopic dermatitis patients, identifying DEGs and performing KEGG and GO analyses, along with correlations between disease severity and itch NRS.

**Results:**

We performed transcriptome profiling on 5 patients with ADP, 6 patients with NADP, and 6 healthy controls. Gene expression analysis revealed significant differences in inflammatory cytokines, suggesting that cytokine-mediated pathways play an important role in the pathogenesis of ADP. GO and KEGG analyses revealed cytokine-cytokine receptor interactions, with Th2 cytokines (SERPINB4, IL4R, IL24) upregulated in ADP and structural repair (BMP2) and metabolic genes (LEPR) elevated in NADP. Severity analysis showed positive correlations with SERPINB4, S100A8, IL24, and TGFB1, and negative correlations with BMP2, IL33, and LEPR. Keratinocyte hyperproliferation and inflammatory genes were commonly upregulated in both ADP and NADP.

**Conclusion:**

These results provide insight into the molecular mechanisms of PN, particularly in the context of atopic dermatitis, and highlight that immune dysregulation and impaired skin barrier function are key factors in pathogenesis.

## Introduction

Prurigo nodularis (PN) is a chronic inflammatory skin disorder characterized by intensely pruritic and hyperkeratotic nodules, predominantly located on the extensor surfaces of the extremities and trunk (1). This debilitating condition significantly impairs quality of life, often leading to severe psychosocial stress due to persistent itching and the appearance of nodules (2, 3). PN is notoriously therapy-resistant; however, the recent FDA approvals of dupilumab and nemolizumab offer promising treatment options (4, 5).

Despite its prevalence, the pathogenesis of PN remains poorly understood, particularly in relation to atopic dermatitis (AD) and the clinical variations observed across racial and ethnic groups. Recent research suggests that PN is driven by a distinct Th22/IL-22-mediated immune response, characterized by elevated IL-22 levels in both systemic circulation and lesional skin (6). CD4^+^ and CD8^+^ T cells play a central role in this pathway, contributing to keratinocyte hyperplasia and disrupted epidermal differentiation, thereby exacerbating pruritus and aligning with PN’s histopathological features (6).

Transcriptomic analyses have also revealed racial differences in immune responses among PN patients. For instance, African American individuals exhibit lower IL-31 upregulation compared to Europeans, who display stronger Th2/IL-13 responses (7). This pattern is supported by the efficacy of nemolizumab in alleviating IL-31-driven pruritus and Th2 inflammation in European PN cohorts, highlighting the importance of personalized treatments tailored to racial and genetic backgrounds (1, 8, 9).

AD significantly contributes to PN development, as both conditions share overlapping immune pathways, including Th1, Th2, and Th17 polarization (9, 10). Mechanisms such as immune-mediated inflammation, bacterial colonization, and impaired skin barrier function are common to both diseases. The upregulation of Th2 cytokines, including IL-13 and IL-4R, underscores this shared immunologic profile (9, 11). The therapeutic success of IL-4R-targeting biologics, such as dupilumab, further supports their common pathophysiology (8, 12–14).

Nonetheless, transcriptomic comparisons between atopic dermatitis-associated prurigo (ADP) and classic AD have revealed significant distinctions (9). ADP exhibits more pronounced Th22 polarization, with higher IL-22 levels and increased expression of IL-22 receptors (IL22RA1 and IL22RA2), which are not commonly elevated in AD (9). ADP also displays neural dysregulation, evidenced by increased nerve fiber density and neuroimmune interactions in lesional skin—features that are less prominent in classic AD (4, 9). In addition, ADP is associated with macrophage activation (M1/M2), increased tumor necrosis factor (TNF) production, fibrosis, tissue remodeling, and angiogenesis, further differentiating it from AD (4, 9, 15). Ultimately, PN is a distinct dermatological entity with both unique and overlapping immunological pathways when compared to AD. While previous studies have primarily focused on distinguishing AD from PN, the transcriptomic differences between ADP and non-atopic dermatitis prurigo (NADP) have not been well characterized. Elucidating these molecular mechanisms may guide the development of more personalized therapeutic approaches and improve clinical outcomes for patients with PN.

## Materials and methods

### Human participants and sample collection

Skin tissue samples were obtained from 17 participants (12 males, 5 females) aged 19 to 75 years, divided into three groups: chronic pruritus without atopic dermatitis (n=6), chronic pruritus with atopic dermatitis (n=5), and normal controls (n=6). *A priori* power analysis (α = 0.05, power = 0.80) indicated that at least 17 participants per group are required. Atopic dermatitis and Prurigo nodularis (PN) were diagnosed by dermatologists based on established criteria. Normal skin samples were collected from the calf region, while PN samples were obtained from affected areas via punch biopsies. Exclusion criteria included immunosuppressive drug use, systemic inflammatory conditions, pregnancy, or breastfeeding.

Samples were collected using sterile techniques, flash-frozen in liquid nitrogen, and stored at -80°C. The study was conducted in accordance with the Declaration of Helsinki and approved by the IRB of Kangnam Sacred Heart Hospital (IRB No. 2022-03-038). Written informed consent was obtained from all participants. Clinical characteristics are summarized in **Table 1**.

**Table 1:** Clinical characteristics of participants.

Characteristics	Normal	Prurigo Patients (Atopic Dermatitis)	Prurigo Patients (Non-atopic Dermatitis)	*P* value
Tissue	6	5	6	
Gender (Female / Male)	2 / 4	2 / 3	1 / 5	0.853
Age (Mean±SD)	30±25.1	54±17.24	63.83±10.87	0.326
Pruritus NRS	0.00±0.00	7±0.63	7±1.15	1
Chronic nodular prurigo (CNPG)	0.00±0.00	3.2±0.75	3.33±0.47	0.452

Values are shown as counts or mean ± SD. P-values were calculated as follows: Gender (Female / Male), χ² test (two-sided); Age, one-way ANOVA across the three groups; Pruritus NRS, Kruskal–Wallis test; CNPG score, Kruskal–Wallis test. Abbreviations: SD, standard deviation; NRS, numeric rating scale; CNPG, chronic nodular prurigo.

CNPG Score: 0: Clear, 1: Almost Clear, 2: Mild, 3: Moderate, 4: Severe

SD: Standard Deviation; Pruritus NRS: Pruritus Numeric Rating Scale

### Transcriptome analysis of skin tissue samples

A total of 17 skin tissue samples were analyzed using Macrogen’s transcriptome sequencing method. RNA was extracted from the skin tissue samples following the manufacturer’s protocol. RNA sequencing was performed on an Illumina HiSeq 2500 platform to generate 100bp paired-end reads. Data preprocessing and quality checks included filtering, logarithm transformation, and normalization, with reproducibility assessed through box plots and density plots. Sequencing reads were aligned to the human reference genome (GRCh38) using HISAT2, and differential expression analysis was conducted using DESeq2. Publicly available transcriptomic data of lesional atopic dermatitis and healthy control skin samples were obtained from the Gene Expression Omnibus [GSE5667 (16) and GSE213849 (17)] and previously published. Platform heterogeneity was corrected by removing batch effects with the *ComBat function* of the *sva package*.

### Principal component analysis

Principal component analysis (PCA) was performed using the DESeq2 package in R to analyze sample variance and clustering. Count data were normalized with variance stabilizing transformation (vst) to adjust for sequencing depth and variability. Genes with low counts (0–10 in fewer than 8 of 17 samples) were filtered out. The vst-transformed data were used for PCA, and results were visualized with ggplot2, with samples categorized as Normal, NADP (Non-Atopic Dermatitis Pruritus), or ADP (Atopic Dermatitis Pruritus). A DESeqDataSet was created with count data and metadata, and DESeq2 was used for differential expression analysis. vst-normalized data were used for PCA, visualized with plotPCA, showing percent variance of the first two components. ggplot2 enhanced the plot with ellipses indicating 95% confidence intervals for each group.

### Differential gene expression analysis

DEG analysis using DESeq2 identified significant genes (padj < 0.05), selecting the top 250 upregulated and downregulated genes by log2FoldChange. Adjusted P-values were calculated with the Benjamini–Hochberg false-discovery-rate procedure. Volcano plots highlighted the top 25 genes, marking significance and fold-change thresholds, while heatmaps visualized expression patterns, including focused views of the top 25 genes. Comparisons (Normal vs. ADP, Normal vs. NADP, NADP vs. ADP) revealed the top 50 DEGs for each group, illustrating the magnitude and significance of expression changes.

### KEGG and GO enrichment analysis

Gene Ontology (GO) and Kyoto Encyclopedia of Genes and Genomes (KEGG) enrichment analyses were conducted using the clusterProfiler package. For GO enrichment analysis, significant genes (padj < 0.05) were annotated using the org.Hs.eg.db database, with the analysis encompassing Biological Process (BP), Cellular Component (CC), and Molecular Function (MF) categories. The results were adjusted using the Benjamini-Hochberg (BH) method, and significant pathways were visualized using dot plots to highlight the top 20 enriched GO terms.

For KEGG pathway enrichment analysis, gene symbols were first mapped to ENTREZ IDs using the bitr function. Mapped genes were analyzed for pathway enrichment in Homo sapiens (hsa) using the enrichKEGG function, with results similarly adjusted using the BH method. Significant pathways were visualized using both dot plots and bar plots. Additionally, specific pathways, such as “hsa04060” (Cytokine-cytokine receptor interaction pathway), were visualized using the pathview package, which generates detailed pathway maps with color-coded gene expression data. This pathway is highly relevant to prurigo nodularis pathogenesis, as it includes key inflammatory mediators (e.g., IL-4, IL-13, IL-24) that play central roles in immune dysregulation and pruritus.

### Correlation analysis of cytokine-cytokine receptor interaction genes with disease severity and pruritus numerical rating scale in patients

Cytokine-cytokine receptor interaction genes from KEGG analysis were correlated with Severity and Pruritus NRS scores. The top 250 upregulated and downregulated genes were log2-transformed and aligned with clinical data. Correlation analyses identified the top 10 genes with the strongest positive and negative correlations for Severity and Pruritus NRS. Scatter plots with regression lines visualized these relationships.

## Results

### Comprehensive genetic and pathway analysis of prurigo nodularis patients with and without atopic dermatitis: insights from GO enrichment and KEGG pathway studies

This study analyzed gene expression in 17 skin transcriptomes, comparing 6 normal individuals and 11 Prurigo Nodularis (PN) patients (5 AD, 6 NAD). **Table 1** presents the demographic and clinical characteristics of the normal and patient groups.

**Figure 1:**
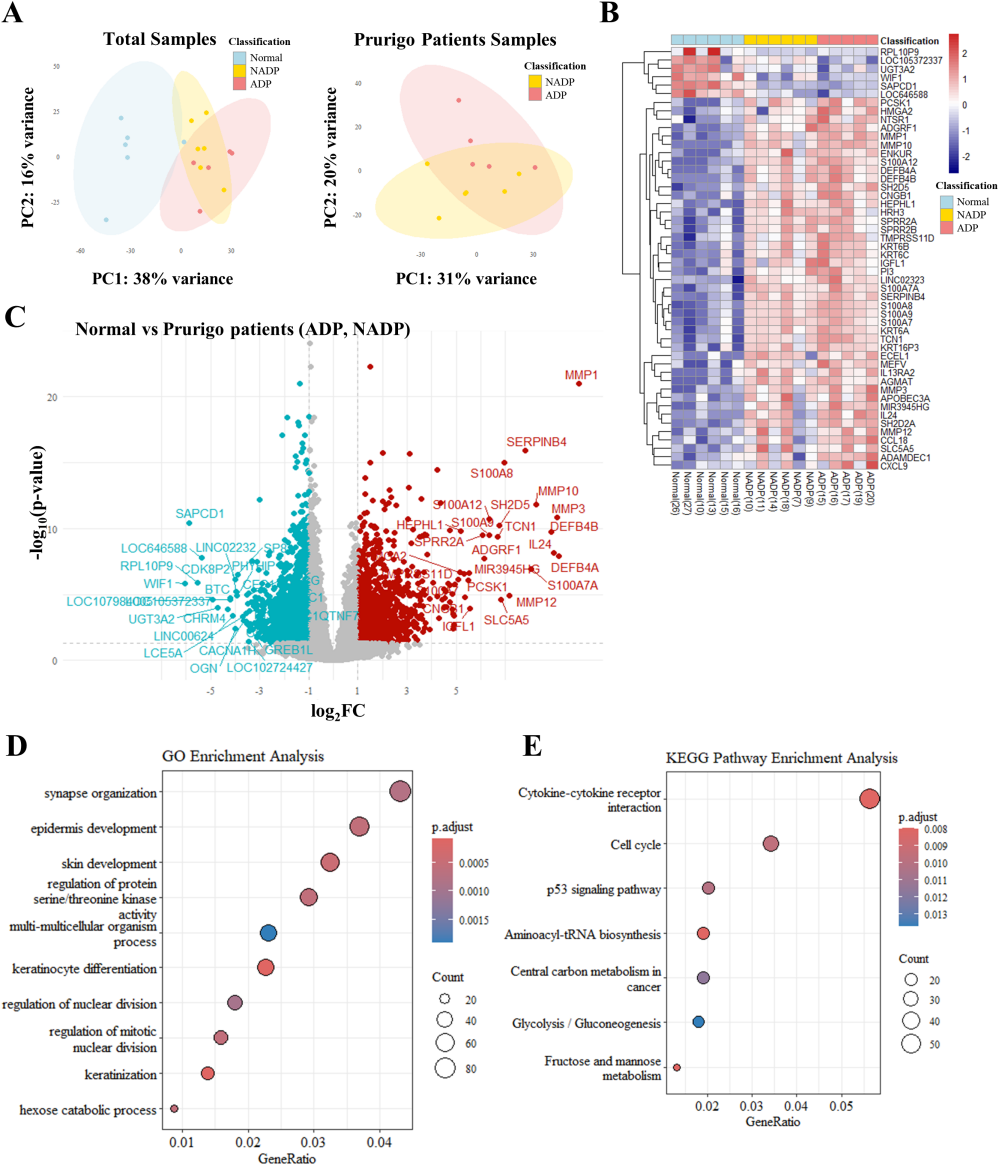
Transcriptomic analysis of skin tissues from healthy controls and patients with Prurigo Nodularis. **(A)** Principal component analysis (PCA) of transcriptomic data. The left plot shows PCA results for skin tissues from healthy controls, non-atopic prurigo nodularis (NADP), and atopic prurigo nodularis (ADP). The right plot shows PCA results comparing NADP and ADP tissues. **(B)** Heatmap of differentially expressed genes (DEGs) among normal, NADP, and ADP tissues, showing the top 50 genes with the most significant expression changes. **(C)** Volcano plot representing DEG analysis between healthy and PN patient tissues. Red dots represent genes that are upregulated in prurigo Nodularis tissues (log2FC ≥ 1 and p-value ≤ 0.05), while teal dots represent genes that are downregulated in prurigo Nodularis tissues (log2FC ≤ -1 and p-value ≤ 0.05). **(D)** Gene Ontology (GO) enrichment and **(E)** KEGG pathway enrichment of DEG patterns identified in normal and prurigo Nodularis tissues.

To evaluate differentially expressed genes (DEGs) between PN patients with and without atopic dermatitis (AD), we performed PCA, which demonstrated clear gene expression differences between normal controls and PN patients, highlighting the influence of AD (**Figure 1A**). A heatmap of the top 50 significant genes (**Figure 1B**) and a Volcano plot (**Figure 1C**) revealed distinct expression profiles among normal controls, PN patients with AD, and those without AD.

GO enrichment analysis indicated associations with epidermal development, keratinocyte differentiation, pruritus, and neurological functions (**Figure 1D**), while KEGG pathway analysis highlighted cytokine-cytokine receptor interactions involving IL-4, IL-13, IL-25, TSLP, and IL-33 (**Figure 1E**, **Supplementary Table 1**). These findings suggest that the top 50 DEGs are closely linked to AD-related pathways and skin inflammation in PN patients.

### Comparative gene expression and pathway analysis in atopic dermatitis prurigo

**Figure 2:**
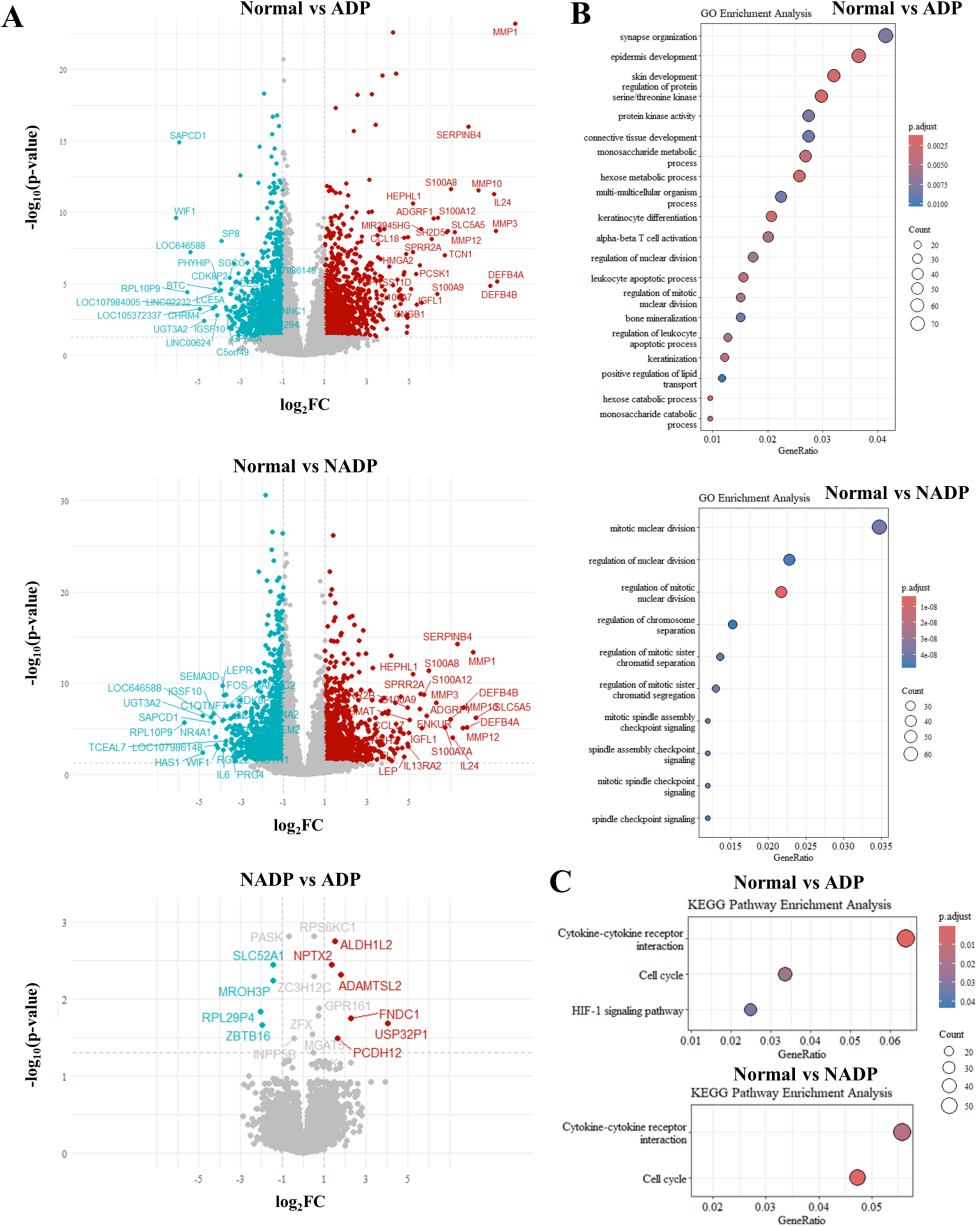
Differential gene expression analysis between Normal, NADP, and ADP tissues. **(A)** Volcano plots showing differential expression analysis for three comparisons: Normal vs. ADP (top), Normal vs. NADP (middle), and NADP vs. ADP (bottom). Red dots indicate genes with significant upregulation (log2FC ≥ 1, p-value ≤ 0.05), while teal dots represent genes with significant downregulation (log2FC ≤ -1, p-value ≤ 0.05). **(B)** GO enrichment analysis based on DEGs from Normal vs. ADP (top) and Normal vs. NADP (bottom). **(C)** KEGG pathway enrichment analysis for DEGs identified in Normal vs. ADP (top) and Normal vs. NADP (bottom).

We analyzed the gene expression differences between Non-Atopic Dermatitis Prurigo (NADP) and Atopic Dermatitis Prurigo (ADP) using a Volcano plot (**Figure 2A**, **Supplementary Table 2**) and performed GO enrichment and KEGG pathway analysis (**Figure 2B**, **Supplementary Table 3**). The functional classification of genes was provided as follows (**Supplementary Table 4–6**). In the NADP/ADP comparison, the upregulated genes of ADP were related to skin and allergic inflammation, while the downregulated genes were related to AD-related inflammation in other tissues, highlighting the importance of AD-related pathways. GO enrichment and KEGG pathway analysis were also performed for the Normal/ADP, Normal/NADP, and NADP/ADP groups (**Figure 2B**). Although the NADP/ADP group had too few genes to obtain meaningful results, the Normal/ADP and Normal/NADP groups were related to epidermal development, keratinocyte differentiation, and synaptic organization in neurological processes, which was consistent with the results of the normal/itch comparison. Additionally, integration with public transcriptomic data (GSE213849) revealed that A total of 128 differentially expressed genes overlapped with GSE213849 and GSE5667. Several genes significantly upregulated in classic AD were also elevated in our ADP samples, indicating shared molecular signatures and reinforcing the immunological overlap between AD and ADP (**Supplementary Figure 1**).

### Distinct gene expression patterns highlighting immune dysregulation and skin barrier dysfunction in Prurigo subgroups

**Figure 3:**
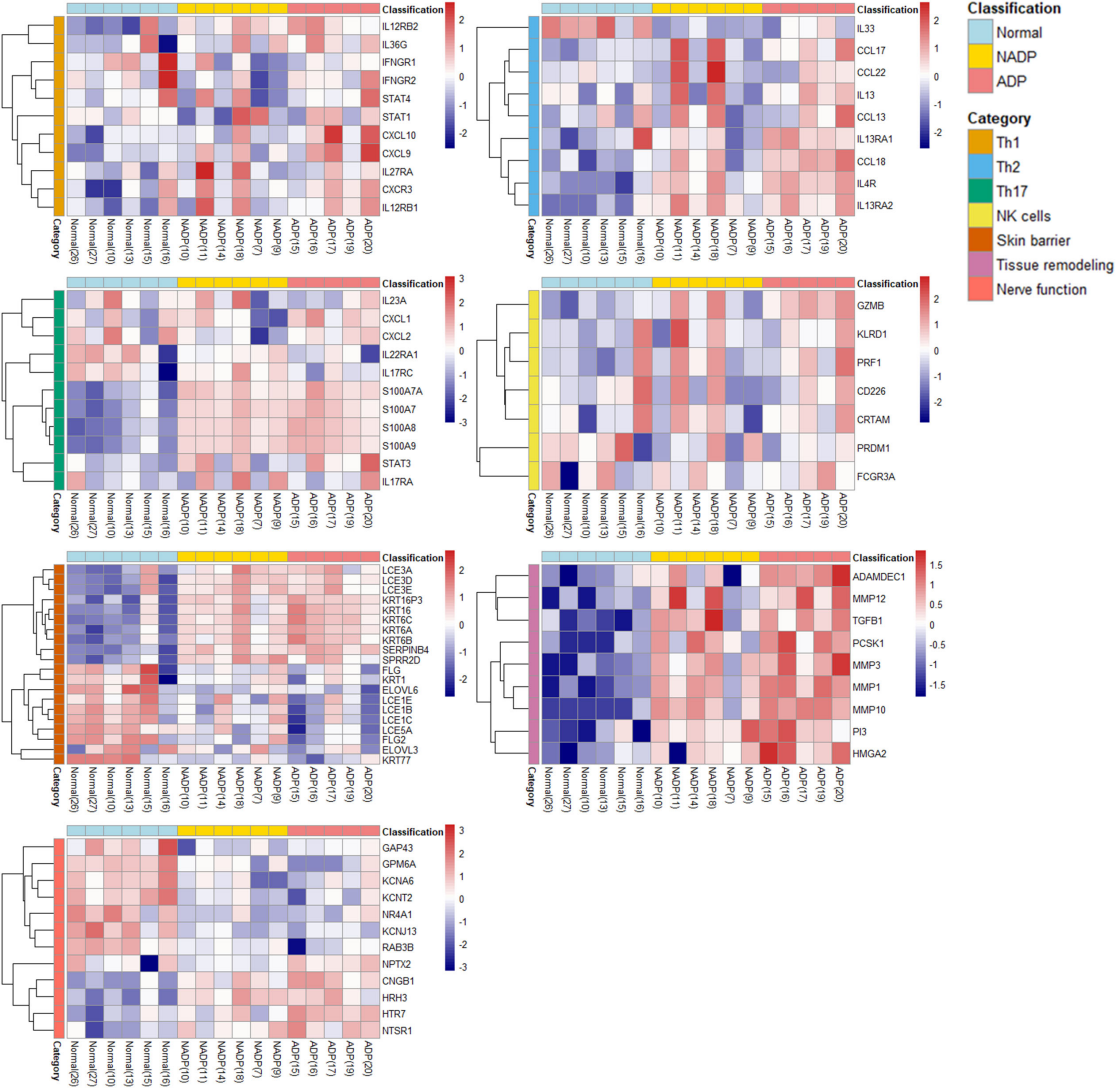
Functional categorization and heatmap analysis. The heatmap displays the expression patterns of DEGs classified into functional categories, including Th1, Th2, Th17, NK cells, skin barrier, tissue remodeling, and nerve function. Gene expression levels are compared across Normal, NADP, and ADP tissues. All DEGs included in the heatmap were selected based on an adjusted p-value (padj) < 0.05 from DESeq2 analysis, indicating statistical significance. Functional grouping was based on known gene functions and prior literature.

These results were analyzed to classify the expression patterns associated with specific genes into various AD-related subgroups (Th1, Th2, Th17, NK cells, Skin barrier, Tissue remodeling, Nerve function, etc.) and identify genes or biomarkers that play important roles in each subgroup. The gene expression analysis in PN patients reveals distinct patterns associated with immune dysregulation and skin barrier dysfunction **Figure 3**. The ADP subgroup shows Th2 inflammation (IL13, IL4R, TGF-β1), skin barrier disruption (LCE3A, KRT16), and tissue remodeling (MMPs), highlighting potential therapeutic targets for personalized treatments. Further supporting our findings, analysis of public microarray data (GSE5667) revealed that genes associated with skin barrier integrity and inflammation, such as SPRR2G and LCE3D, were upregulated in both AD and our ADP dataset (**Supplementary Figure 2**).

### Correlation analysis of severity and NRS in atopic dermatitis: inflammation and immune regulation insights

**Figure 4:**
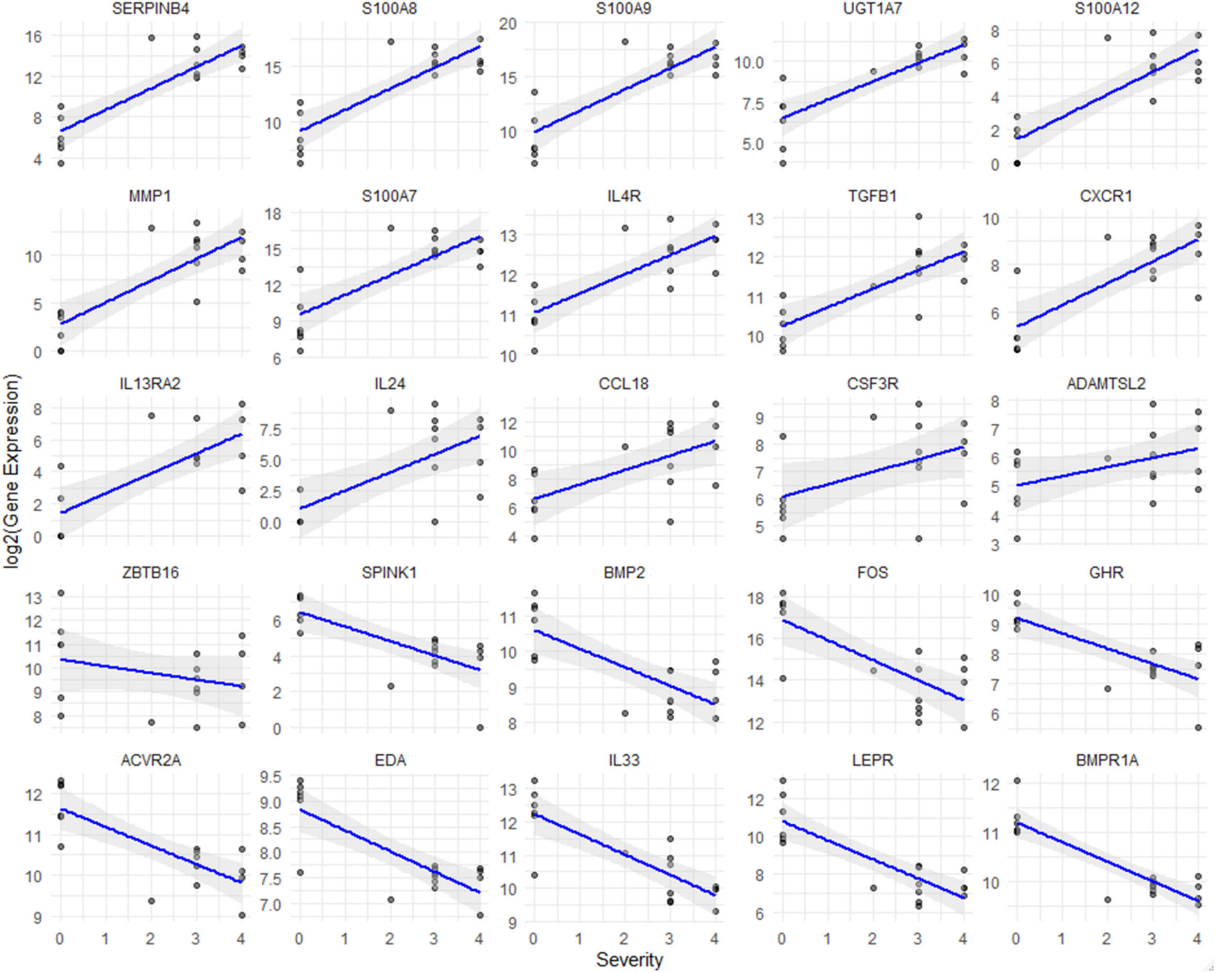
Correlation analysis between gene expression and severity of prurigo nodularis. Scatter plots show the relationship between gene expression levels and chronic nodular prurigo severity scores (0-4). The x-axis represents the severity score, while the y-axis indicates log2-normalized FPKM values for each gene. The blue line represents the regression trend, and the shaded grey area indicates the confidence interval. All genes shown in the plots demonstrated statistically significant correlations with severity (Spearman correlation, p < 0.05).

We conducted a correlation analysis between prurigo severity and pruritus (as measured by the NRS) and gene expression levels in ADP (**Figure 4**). Genes such as SERPINB4, S100A8, IL24, and TGFB1 positively correlate with disease severity, suggesting their involvement in inflammation and immune responses in ADP. Conversely, genes like BMP2, IL33, and LEPR show negative correlations. S100 family genes and CCL18 further highlight their role in inflammation, offering potential therapeutic targets for managing ADP. These results suggest that gene expression patterns vary with disease severity and that specific genes are associated with the progression of ADP, depending on whether their expression levels are increased or decreased.

### Differential gene expression in atopic and non-atopic Prurigo highlights shared and unique pathways in inflammation and tissue remodeling

**Figure 5:**
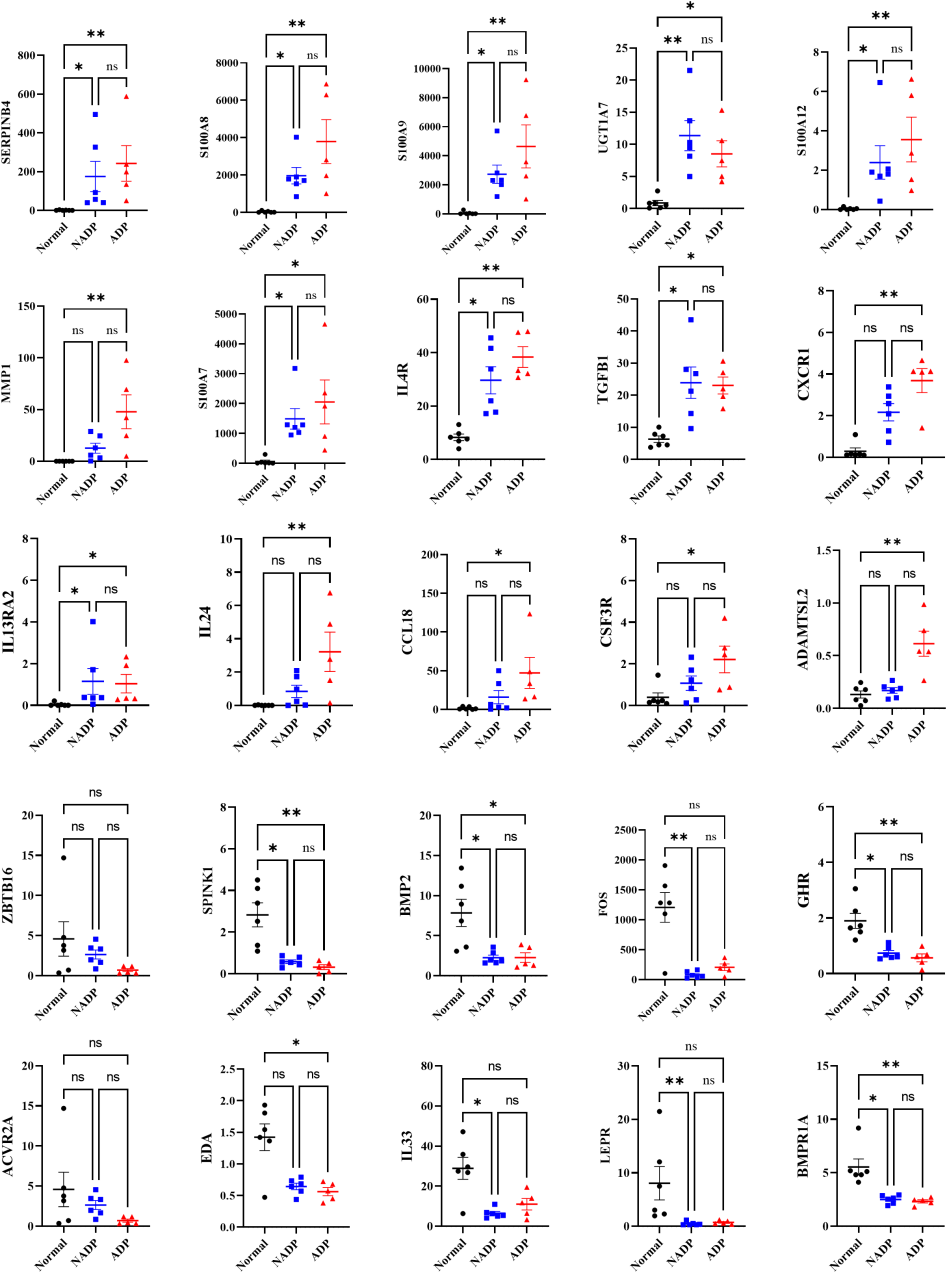
Gene expression levels of core genes identified as potential biomarkers in prurigo nodularis. The graphs display the expression levels of core genes identified as potential biomarkers for distinguishing between disease groups in prurigo nodularis. The y-axis represents FPKM values, with black dots indicating the Normal group, blue dots indicating the NADP group, and red dots indicating the ADP group. Error bars represent the mean ± standard deviation. Asterisks denote statistical significance (*p ≤ 0.05, **p ≤ 0.01).

Gene expression analysis of ADP and NADP revealed that ADP has higher expression of inflammatory and immune-regulating genes (e.g., SERPINB4, S100 family, IL4R, IL24), while NADP shows relatively lower expression of inflammatory markers and distinct expression patterns in structural repair (e.g., BMP2) and metabolic regulation genes (e.g., LEPR). Notably, although BMP2 and LEPR were initially considered elevated in NADP, **Figure 5** indicates these genes are in fact downregulated compared to healthy controls. These differences highlight potential pathway divergence and suggest that a Th2-mediated immune response is particularly prominent in ADP, supported by the upregulation of SERPINB4, S100A8, IL4R, and IL24. Targeting Th2 cytokines may therefore offer effective therapeutic strategies (**Figure 5**). Moreover, genes such as TOP2A and SELL, previously identified as significantly upregulated in PN compared to healthy controls (GSE213849), were also elevated in our ADP samples, suggesting a shared transcriptional landscape between ADP and classical PN (**Supplementary Figure 3**).

### Differential gene expression and pathway analysis in atopic dermatitis: insights into inflammation, keratinocyte hyperproliferation, and immune dysregulation

Our study identified 415 genes uniquely upregulated in ADP, 412 in NADP, and 836 in both groups, while 840 were commonly downregulated **Table 2**. Genes linked to keratinocyte hyperproliferation (MMP1, S100A8, S100A9) and inflammation (IL24, CXCL9) were upregulated in both groups **Table 2**. whereas downregulated genes like WIF1 and CHRM4 highlighted distinct pathways. Upregulated fibrosis and tissue remodeling genes (MMP3, MMP12) suggest potential therapeutic targets to improve AD treatment strategies.

**Table 2:** Functional categorization of differentially expressed genes in PN patients.

Functional categorization	Gene
Keratinocyte hyperproliferation	MMP1, SPRR2A, S100A8, S100A9 ↑ WIF1 ↓
Fibrosis and tissue remodeling	MMP1, MMP3, MMP10, MMP12 ↑ LEPR, IGSF10 ↓
Pruritus and neurogenic inflammation	IL24, MMP1, NTSR1, HRH3, HTR7 ↑ CHRM4, KCNJ13 ↓
Chronic inflammation (Th1/Th2/Th17/Th22)	IL24, CXCL9, CXCL10, IL4R, IL13RA2, S100A8, S100A9, SERPINB4 ↑ NR4A1 ↓
Innate immunity	DEFB4A, S100A8, S100A9 ↑

## Discussion

This study presents a comprehensive gene expression analysis of PN patients, differentiating between Atopic dermatitis Prurigo (ADP) and Non-Atopic dermatitis Prurigo (NADP) subtypes and comparing them with healthy controls. Our results revealed distinct transcriptomic differences not only between PN patients and healthy controls but also between ADP and NADP subgroups, underscoring the importance of understanding genetic and molecular distinctions within PN for developing targeted therapies.

PCA revealed distinct gene expression profiles between PN patients and normal controls, with differences between ADP and NADP. GO and KEGG analyses identified unique immunological features, including upregulated cytokine-cytokine receptor interactions. In particular, particularly the Th2 pathway involving IL-4, IL-13, IL-33, and TSLP. This is consistent with previous findings that both ADP and AD share Th2 polarization (9). Previous studies demonstrated the efficacy of Th2-targeting therapies, such as dupilumab, in treating PN (8, 12–14), which aligns with findings from Japanese cohorts identifying Th2 as a major driver in PN among Asian populations (18, 19).

Our study examined the Th22/IL-22 pathway, which is known to disrupt the skin barrier and exacerbate pruritus in atopic dermatitis. IL-22 promotes keratinocyte proliferation and impairs barrier function; however, in our dataset, Th22-related gene expression, including IL-22 and its receptors (IL22RA1, IL22RA2), was not significantly elevated in either the ADP or NADP groups, as also reflected in **Figures 3**-**5**. This finding suggests that the Th22 axis is not a dominant immune pathway in our Asian PN cohort, aligning with previous transcriptomic studies in similar populations (6). This contrasts with African-American or European populations, where IL-22 and other Th22 cytokines are often upregulated and play a central role in disease pathology (6). These observations underscore racial and ethnic variations in immune activation pathways, with lower Th22 activity but relatively heightened Th2-mediated responses in Asian patients, which may have important implications for tailored therapeutic strategies.

IL-31, a cytokine strongly linked to pruritus and inflammation, is known to stimulate cutaneous nerve fibers and drive neuroimmune activation in prurigo nodularis (PN) patients (20). However, in our current transcriptomic analysis of lesional skin samples, IL-31 expression was not significantly elevated in either the ADP or NADP groups compared to healthy controls. This finding is consistent with previous reports in Asian PN cohorts, where IL-31 expression was either low or absent at the transcriptomic level (6, 20). One possible explanation for this discrepancy lies in racial or ethnic differences in immune signaling, with IL-31-mediated pathways more prominently observed in European populations, while IL-17 or IL-22-related pathways appear to be more dominant in Asian patients (6, 21). Additionally, the tissue specificity of IL-31 expression—more frequently detected in serum or peripheral blood mononuclear cells than in skin tissue—may also contribute to the lack of differential expression in our skin biopsy data (11). These observations underscore the complex and heterogeneous pathogenesis of PN and highlight the need for integrative analyses combining transcriptomic, proteomic, and serum-based cytokine profiling in future studies.

For pathway analysis, GO enrichment and KEGG assessments on normal/ADP, normal/NADP, and NADP/ADP groups revealed prominent pathways. Epidermal development and synaptic organization were highly significant in ADP compared to normal controls, and cytokine-cytokine receptor interaction pathways were relevant for both ADP and NADP. The IL-17 signaling pathway, in particular, was more active in NADP compared to controls, paralleling findings in psoriasis, where IL-17 is known to play a central role (6). These findings highlight the IL-17A-Endothelin-1 axis as a potentially important driver in PN pathogenesis (21).

Comparative analysis of gene expression between ADP and NADP further clarified pathophysiological distinctions, revealing upregulation of genes such as NPTX2, ADAMTSL2, USP32P1, FNDC1, PCDH12, and ALDH1L2 in ADP. NPTX2, associated with chronic pruritus, is highly expressed in sensory neurons (22), while FNDC1 in fibroblasts (23), and PCDH12, a marker for MrgprA3+ neurons linked to itch perception (24) were more pronounced in ADP. The upregulation of genes related to neural function and tissue remodeling in ADP suggests that neuroregulatory dysfunction and fibroproliferative tissue remodeling may be more pronounced in ADP than NADP.

Fibrosis is central to PN pathology, marked by increased keratinocyte proliferation, ECM remodeling, and tissue thickening. Upregulated MMPs (e.g., MMP1, MMP3, MMP12) drive ECM breakdown and fibrotic nodule formation. Similarly, SPRR2A, associated with hyperproliferative (25) and reparative responses (26–28), and decreased IGSF10 expression may exacerbate uncontrolled fibrosis, contributing to the increased dermal thickness typical of nodular prurigo (27).

Among commonly upregulated genes in both ADP and NADP, SERPINB4—induced by IL-22, IL-17, IL-4, and IL-13—correlates with disease severity, highlighting its potential as a biomarker for treatment efficacy. IL-24, another promising therapeutic target, exacerbates pruritus and inflammation, particularly in response to *Staphylococcus aureus* (28). Antimicrobial peptide DEFB4B, critical in skin barrier integrity, shows decreased expression in PN, linking it to susceptibility to infection (29). IGFL1, which supports epithelial proliferation, and SPRR2A (25), crucial for keratinocyte differentiation, emerge as targets for enhancing skin recovery and preventing disease progression in PN and AD (30). Upregulated IL4R, CCL18, and TGFB1 indicate shared Th2-driven inflammation in ADP and NADP, while ADP-specific genes like CSF3R and ADAMTSL2 highlight heightened inflammation, remodeling, and neuroimmune interactions.

Single-cell RNA-sequencing in PN and AD reveals fibroblasts in AD lesions expressing CCL2, CCL19, and CCL11, which drive immune cell recruitment and inflammation via the IKKβ/NF-κB pathway, worsening AD pathology (31). Comparative studies between PN and AD have shown that both conditions exhibit type 2 immune bias, but AD showed extensive immune activation of CD8A+IL9R+IL13+ cytotoxic T cells (32). In contrast, PN is characterized by extracellular matrix remodeling, collagen synthesis, and fibrosis, and has been shown to have CXCL14-IL24+ fibroblasts (33). In addition, a recent study identified a population of COL6A5+COL18A1+ fibroblasts that are present exclusively in AD lesions (34). Our study found significant upregulation of IL-4R, IL-13R, and IL-24 in AD lesions, contributing to Th2 responses. KEGG analysis revealed cytokine-cytokine receptor interactions as key to PN and AD progression. Genes like S100 family, CCL18, and CSF3R increased with AD severity, linking gene expression to disease progression and identifying therapeutic targets for inflammation and itching. Correlation analysis between the severity of PN with the top 500 significant genes revealed a positive correlation for IL4R, IL13RA2 (Th2), TGFB1 (fibrosis in PN), CXCR1 (chemoattractant), and IL24 (Th2). Similarly, a positive correlation with pruritus NRS was observed for IL4R, CXCR1, TGFB1, IL13RA2. These findings suggest that these cytokines and their receptors could serve as potential therapeutic targets. Additionally, KEGG pathway analysis identified upregulation of ADP-related DEGs such as CLCF1, CXCL9, and CXCL10, suggesting that the CXCL9/CXCL10 axis may play a key role in pruritic neuroinflammation (35). Increased expression of IL24, S100A8, and S100A9 contributes to neuroinflammation, thereby activating sensory neurons that induce itch. MMP1 degrades extracellular matrix components, exposing the nerves to inflammatory mediators, thereby increasing nerve sensitivity. Conversely, decreased expression of CHRM4 and KCNJ13 may impair sensory nerve signaling, thereby increasing nerve excitability and altering itch perception.

Several genes, including BMPR1A, EDA, IL33, and BMP2 were notably downregulated in PN. BMPR1A regulates skin inflammation via key signaling pathways (36, 37). while EDA is associated with cell death, differentiation, and migration processes (38). In this study, IL-33 levels were found to be reduced in PN and negatively correlated with pruritus severity (6, 39, 40). A potential hypothesis is that while IL-33 is a key driver of allergic inflammation (39–43), it also plays an essential role in promoting re-epithelialization (43–45), inducing ILC2 proliferation (46, 47), facilitating wound healing (43–45, 47) and supporting the induction and maintenance of regulatory T cells (Tregs) (48–50). Reduced IL-33 expression may impair wound healing, preventing the resolution of scratch-induced lesions and promoting the transition from acute to chronic inflammation (39–42, 51, 52).

BMP2, which plays a role in cell growth, maturation, and fibrotic proliferation across the dermis and epidermis, was also downregulated (53). Decreased BMP2 and related genes in PN indicate a shift to chronic fibrosis, while downregulated LEPR and GHR in ADP suggest systemic dysfunction. Targeting these and structural repair pathways may offer holistic therapies.

In conclusion, this study elucidates critical molecular and immunological differences between ADP and NADP, providing insights into distinct inflammatory pathways in each subtype. Pathways such as IL-17 in NADP and neuroregulatory dysfunction in ADP highlight the potential for personalized treatments, particularly by targeting cytokines such as IL4R, IL13RA2, and TGFB1, which are associated with disease severity. The influence of racial and ethnic factors further emphasizes the need for individualized therapies based on molecular characteristics, warranting further research to refine treatment strategies for PN’s diverse subtypes.

## Data Availability

The RNA-seq datasets generated and analyzed for this study involve human participants and are therefore not publicly available due to ethical and privacy restrictions. Requests to access the datasets should be directed to the corresponding author.
