# Supplementary material for: Transcriptomic analysis of skin biopsies in Prurigo nodularis patients: with and without atopic dermatitis
Source: Front Immunol. 2025 Nov 5;16:1572413. doi: 10.3389/fimmu.2025.1572413 (PMC12627025; doi:10.3389/fimmu.2025.1572413)
Supplement: Supplementary file 1 [file DataSheet1.docx]

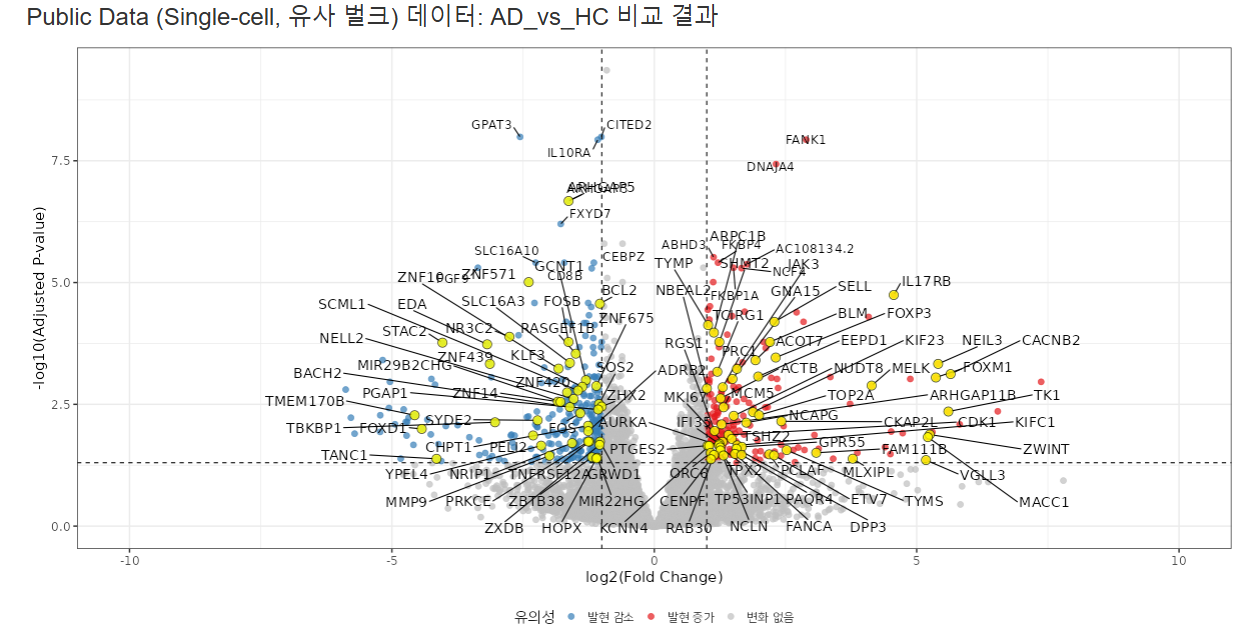


**Supplementary figure 1.** Volcano plot showing differentially expressed genes (DEGs) between atopic dermatitis (AD) and healthy controls (HC) based on public transcriptomic datasets (GSE213849, integrated single-cell and bulk RNA-seq; total n = 16: HC = 5, AD = 5, PN = 6)).

The x-axis indicates the log₂ fold change in gene expression (AD vs. HC), and the y-axis represents the −log₁₀ of the adjusted p-value. Genes significantly upregulated in AD are shown in red, and downregulated genes in blue, based on thresholds of |log₂FC| ≥ 1 and adjusted p < 0.05. Yellow dots represent genes that were also found to be significantly upregulated in our own ADP vs. Normal transcriptomic dataset, highlighting shared molecular signatures between public AD data and our ADP cohort. Non-significant genes are shown in gray. Several representative genes (e.g., IL10RA, FOXM1, MMP9, CDK1) are labeled. Vertical and horizontal dashed lines denote the log₂ fold-change and significance thresholds, respectively.


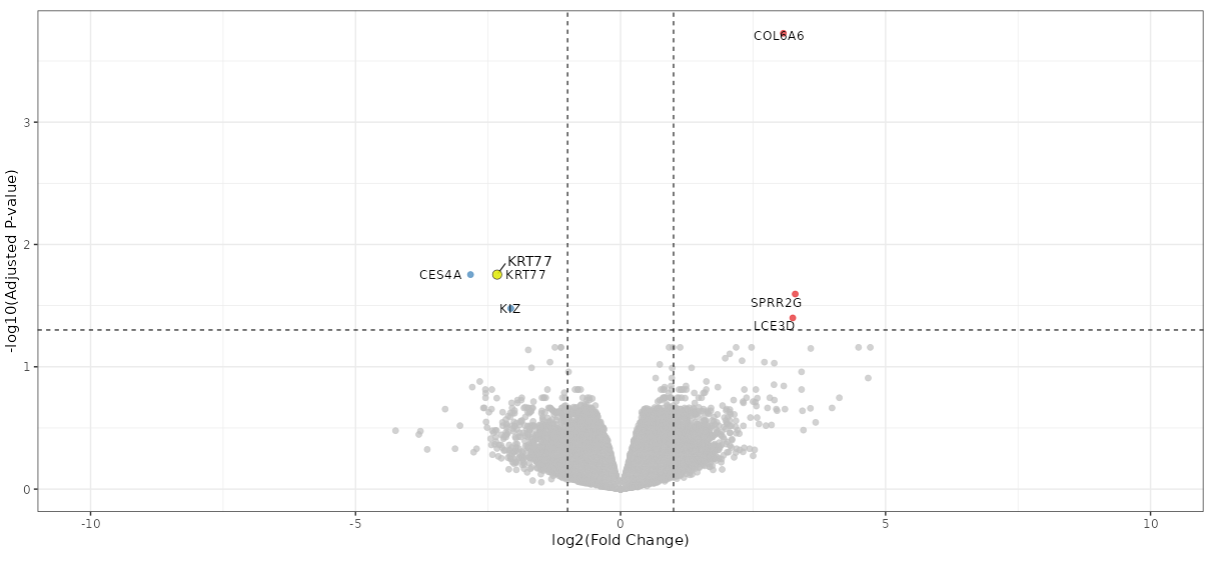


**Supplementary figure 2.** Volcano plot showing differentially expressed genes (DEGs) between atopic dermatitis (AD) patients and healthy controls (HC) based on public microarray data (GSE5667) of skin tissue samples (total n = 11: HC = 5, LAD = 6).

The x-axis represents the log₂ fold change in gene expression (AD vs. HC), and the y-axis shows the −log₁₀ of the adjusted p-value. Genes significantly upregulated in AD are marked in red, and downregulated genes in blue, based on a threshold of |log₂FC| ≥ 1 and adjusted p < 0.05. Yellow dots indicate genes that overlapped with those significantly upregulated in our own ADP (atopic dermatitis prurigo) dataset, emphasizing shared transcriptional signatures between public AD data and the ADP cohort analyzed in this study. Labeled genes such as COL8A6, SPRR2G, LCE3D, and KRT77 represent notable changes relevant to skin barrier function and inflammation. Dashed lines denote the thresholds used for fold change and statistical significance.


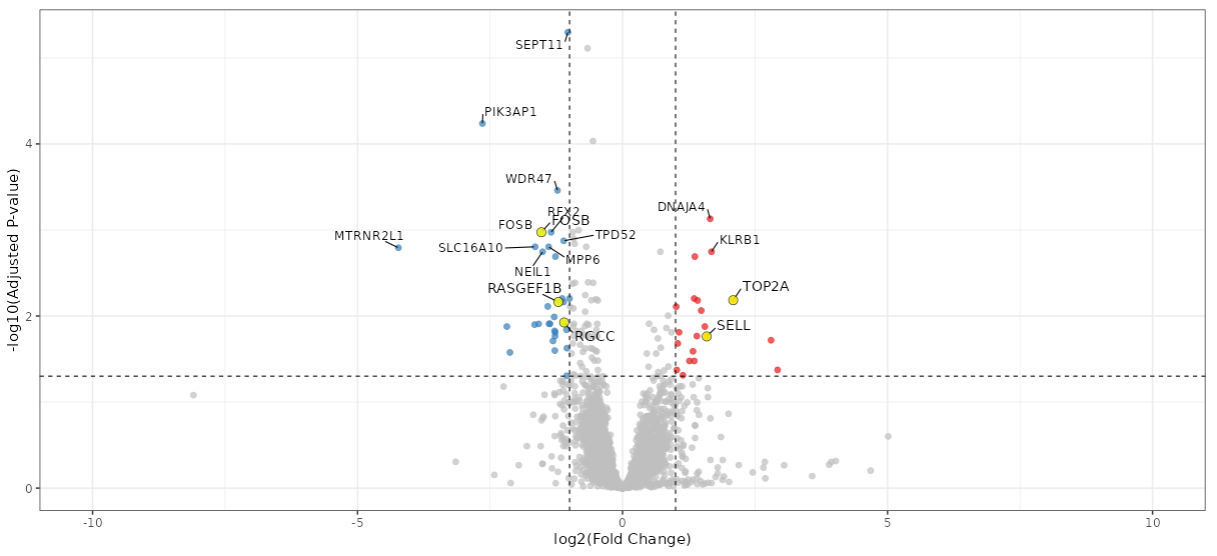
**Supplementary figure 3.** Volcano plot showing differentially expressed genes (DEGs) between prurigo nodularis (PN) patients and healthy controls (HC), based on integrated public transcriptomic data (GSE213849, single-cell and bulk RNA-seq).

The x-axis indicates the log₂ fold change (PN vs. HC), and the y-axis represents the −log₁₀ of the adjusted p-value. Genes significantly upregulated in PN are colored in red, and downregulated genes are shown in blue, using thresholds of |log₂FC| ≥ 1 and adjusted p < 0.05.

Yellow-highlighted genes represent DEGs that also overlapped with our ADP (atopic dermatitis prurigo) vs. normal transcriptomic results, indicating shared transcriptional signatures between ADP and classic PN.

Labeled genes such as TOP2A, SELL, FOSB, and MPP6 reflect immune regulation, cell proliferation, and structural remodeling pathways relevant to PN pathogenesis. Vertical and horizontal dashed lines mark the cutoffs for fold change and statistical significance, respectively.
