# Supplementary figures and images for: Transcriptomic analysis of skin biopsies in Prurigo nodularis patients: with and without atopic dermatitis
Source: Front Immunol. 2025 Nov 5;16:1572413. doi: 10.3389/fimmu.2025.1572413 (PMC12627025; doi:10.3389/fimmu.2025.1572413)

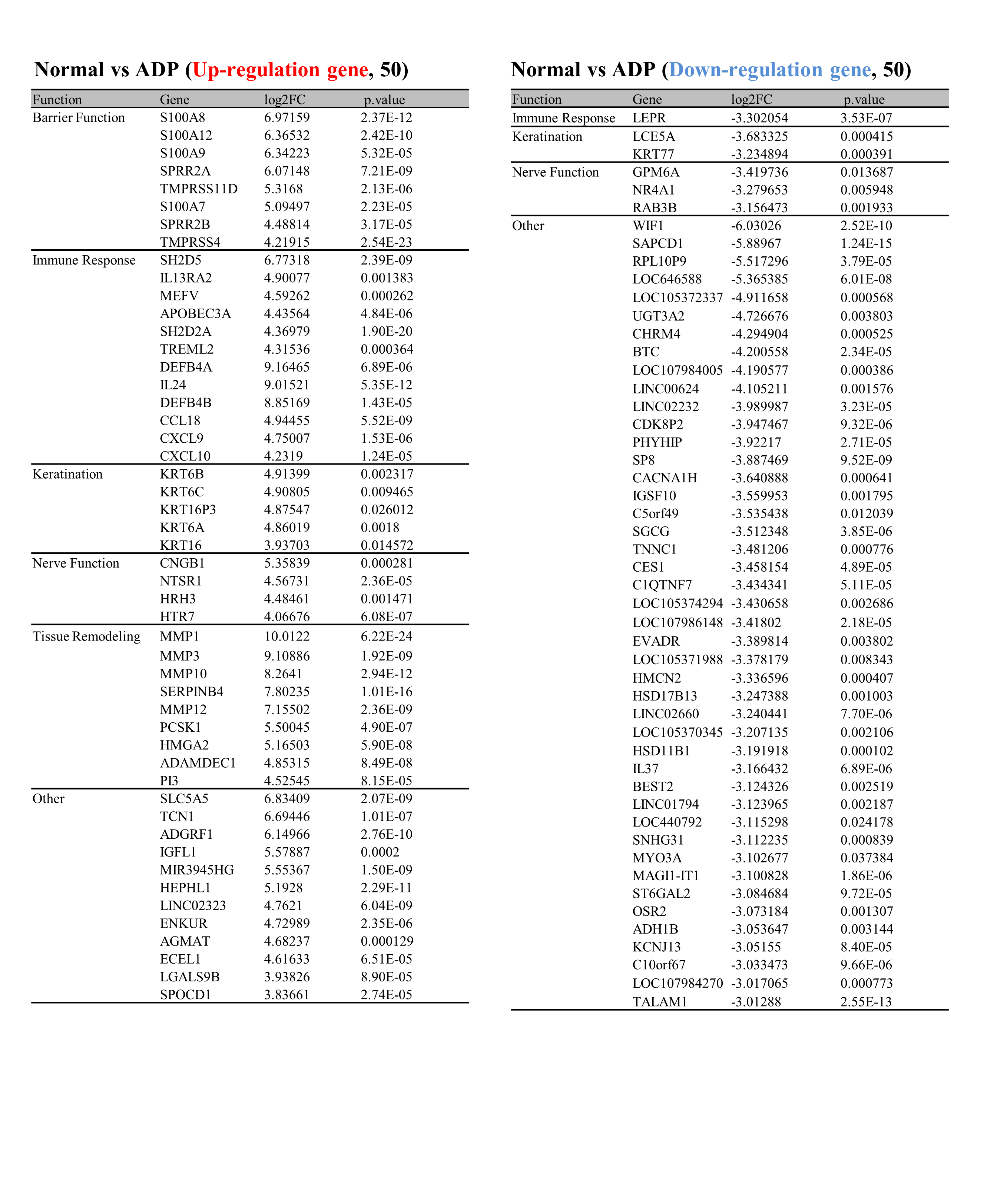

Supplement: Supplementary file 2 [file Image1.tif]

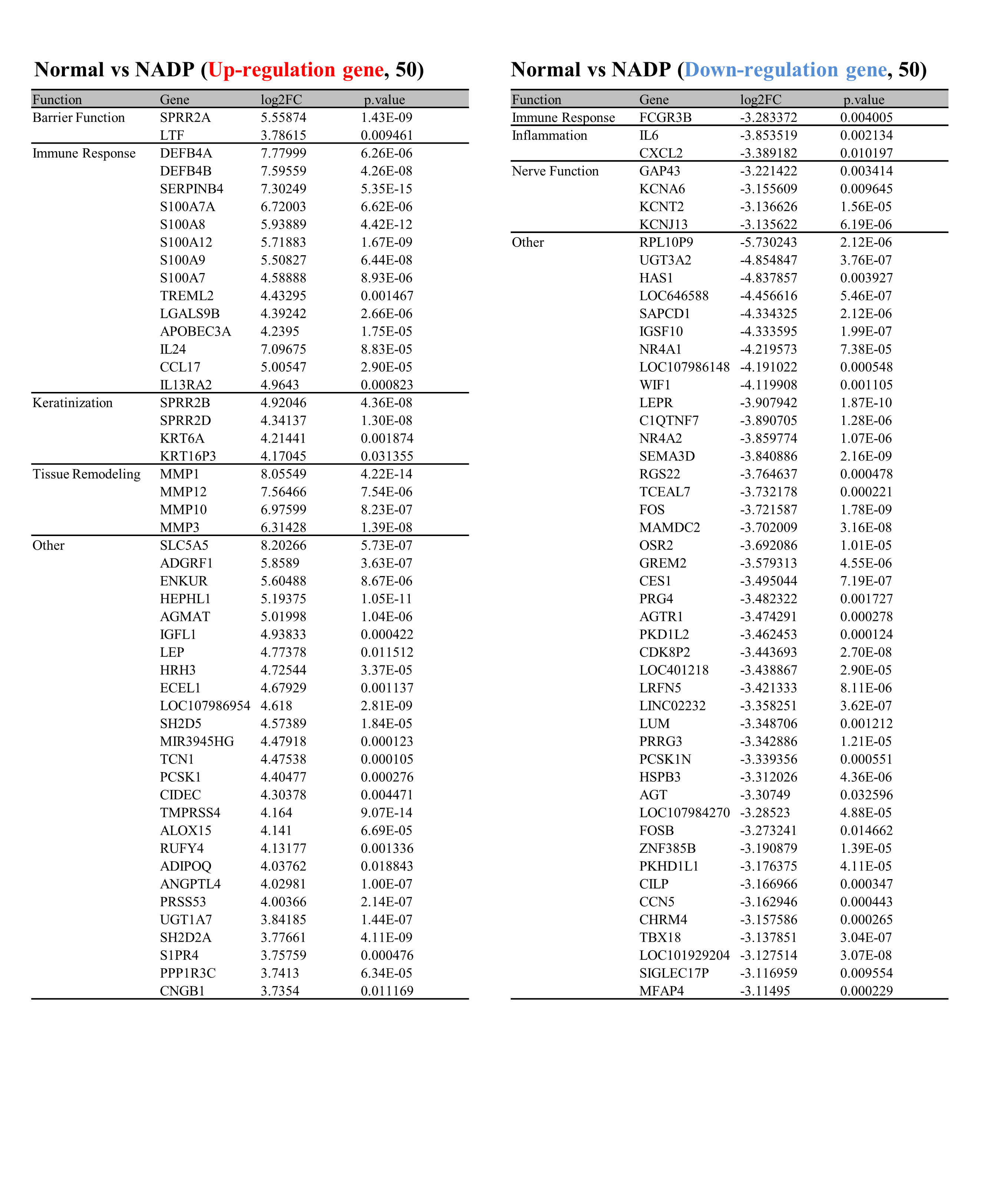

Supplement: Supplementary file 3 [file Image2.tif]

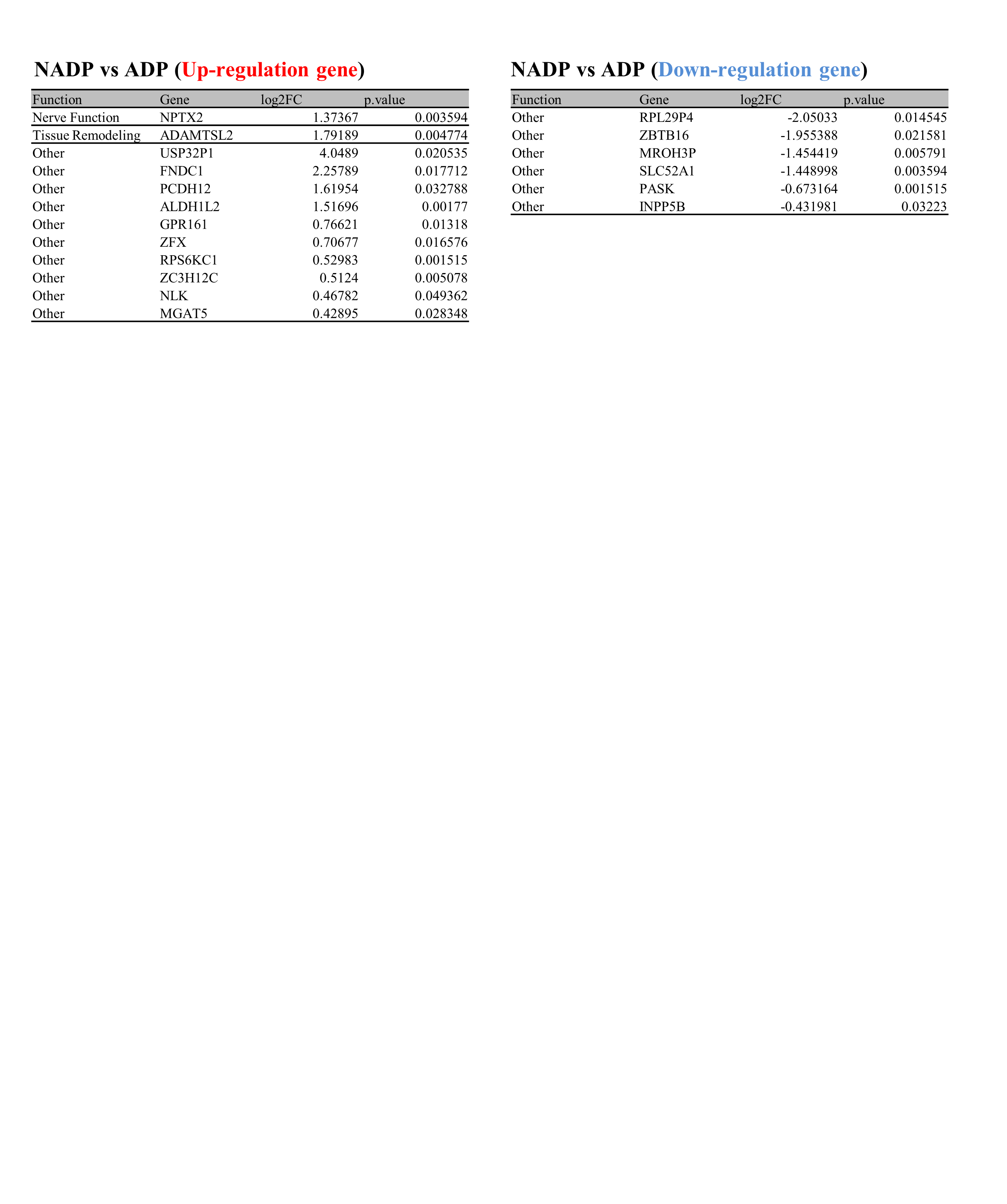

Supplement: Supplementary file 4 [file Image3.tif]
